# A GSH-responsive nanophotosensitizer for efficient photodynamic therapy†

**DOI:** 10.1039/c8ra08549h

**Published:** 2018-12-19

**Authors:** Wei Pan, Mingwan Shi, Yanhua Li, Yuanyuan Chen, Na Li, Bo Tang

**Affiliations:** College of Chemistry, Chemical Engineering and Materials Science, Key Laboratory of Molecular and Nano Probes, Ministry of Education, Collaborative Innovation Center of Functionalized Probes for Chemical Imaging in Universities of Shandong, Institute of Molecular and Nano Science, Shandong Normal University Jinan 250014 P. R. China lina@sdnu.edu.cn +86-531-86180017

## Abstract

Photodynamic therapy (PDT) is a promising cancer treatment modality, which depends on the reactive oxygen species (ROS) generated by a photosensitizer to kill cancer cells. The lack of selectivity and the over-production of glutathione (GSH) in cancer cells are the two major challenges for efficient and safe cancer PDT because they can cause harm to normal tissues and eliminate ROS in cancer cells. Herein, we report a GSH-responsive nanophotosensitizer based on CoOOH nanosheets for PDT of cancer. The nanophotosensitizer shows negligible photo-toxicity toward normal cells because of the quenching effect between CoOOH and photosensitizer Ce6. In the presence of overexpressed GSH, Ce6 molecules can be released into cancer cells because of GSH induced degradation of CoOOH nanosheets. *In vivo* experiments demonstrated that the tumor growth was efficiently inhibited by the CoOOH-based PDT strategy. The current nanophotosensitizer represents a promising smart platform to synergistically improve the therapeutic index and safety of PDT.

## Abstract 2

A GSH-responsive nanophotosensitizer based on CoOOH nanosheets was developed for the photodynamic therapy of cancer.

## Graphical abstract

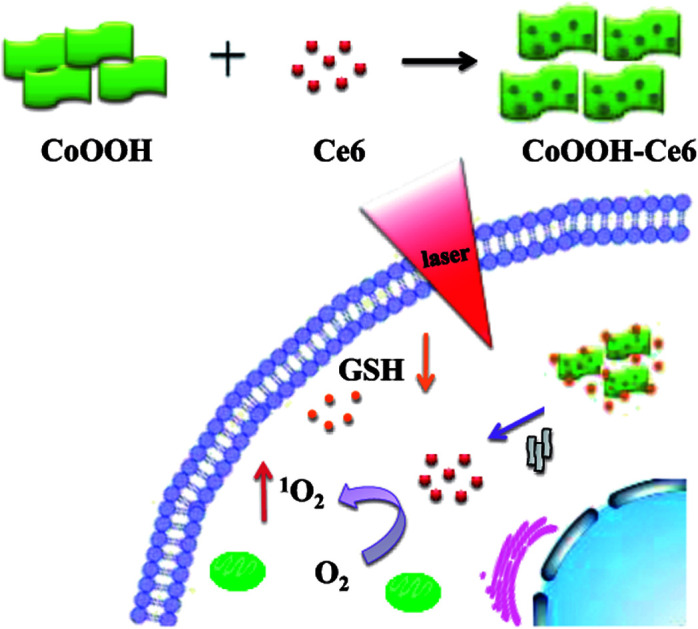

## Introduction

Malignant tumors are one of the major chronic diseases that seriously threaten human health.^1,2^ Creating a good strategy to treat cancer cells has attracted increasing attention during the past decades.^3^ Photodynamic therapy (PDT) has been one of the most attractive cancer treatment strategies owing to its excellent controllability, short treatment cycle and low dark toxicity.^4–10^ The major mechanism of PDT is suggested to be the generation of reactive oxygen species (ROS), due to the energy transfer between the excited photosensitizer and the nearby oxygen molecules, which can further denaturalize the biomolecules in cancer cells.^11–20^ However, it is reported that the generated ROS can be removed by superoxide dismutase (SOD) and catalase (CAT), *etc.*^21^ Moreover, the overexpressed glutathione (GSH) in cancer cells can directly react with ROS and further prevent them from oxidation stress.^22,23^ Notably, the generated ROS is even more toxic to normal cells due to the lower GSH concentration and higher oxygen content, which resulted in the side effects of PDT.^24–26^ Therefore, it is highly desired to develop a safe and efficient PDT platform for PDT with enhanced selectivity and reduced side effects.

Cobalt oxyhydroxide (CoOOH) nanosheet, a kind of 2D nanomaterial, has been widely used in the biological fields because of its easy preparation, functionalization and high biocompatibility.^27,28^ Moreover, CoOOH nanosheets have a large specific surface area, which makes it suitable for drug loading and substrate detection.^29,30^ Most importantly, the optical quenching properties of CoOOH makes it an ideal platform for photosensitizer delivery and responsive cancer treatment, which can significantly reduce the toxicity of photosensitizer before reaching target sites. After CoOOH reacted with cancer cell-overexpressed antioxidant GSH, the CoOOH will be collapsed and the quenched photosensitizer can be reactivated, which is helpful to synergistically enhance the safety and efficiency of PDT due to the selective activation and GSH elimination.

**Scheme 1:**
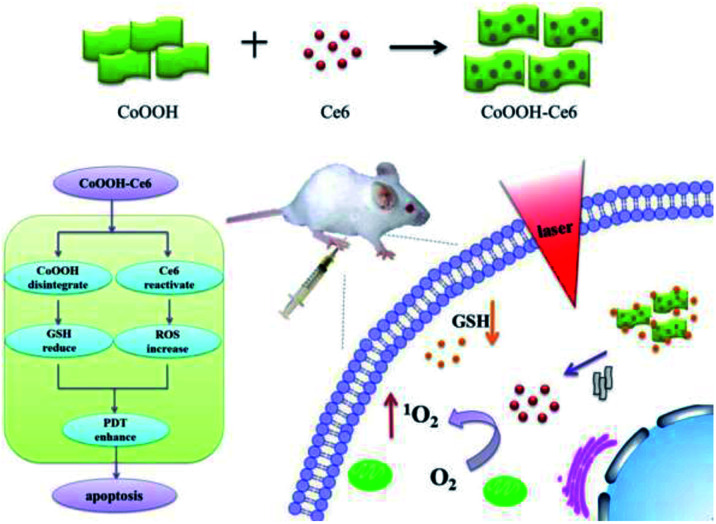
Schematic illustration of the synthesis of the CoOOH–Ce6 and photodynamic therapy in the cell with nanophotosensitizer.

On this basis, we designed and synthesized a CoOOH-based nanophotosensitizer as a smart PDT platform for cancer PDT. The nanophotosensitizer was synthesized by directly decorating photosensitizer chlorin e6 (Ce6) onto CoOOH nanosheets through an amide linkage between the amino group and the carboxyl group (as shown in Scheme 1). In this nanophotosensitizer, CoOOH plays two important roles, one is the carrier with quenching capacity, which can deliver Ce6 molecules into cancer cells while prevent the generation of ^1^O_2_ in normal cells. The other is as an oxidant that can reduce the intracellular GSH. When the nanophotosensitizer was injected into the tumor site, the reaction of CoOOH nanosheets with intratumoral GSH resulted in the degradation of CoOOH and the release of Ce6. And then, the reduction of intracellular GSH concentration resulted lower resistance of cancer cells toward ^1^O_2_, so the tumor cells can be effectively killed under the laser irradiation. Therefore, we believe that the current nanophotosensitizer should be a promising candidate for smart cancer therapy with enhanced efficiency and selectivity.

## Experimental

### Materials and instruments

Cobalt chloride hexahydrate (CoCl_2_·6H_2_O) was purchased from Tianjin Guangfu (Tianjin, China). *N*,*N*-Dimethylformamide (DMF), sodium hypochlorite (NaClO), sodium hydroxide (NaOH) were purchased from China National Pharmaceutical (Shanghai, China). 3-(4,5-Dimethylthiazol-2-yl)-2,5-diphenyltetrazolium bromide (MTT) and GSH were purchased from Sigma Aldrich (U.S.). 3-Aminopropyltriethoxysilane (APTES) was purchased from Alfa Aesar (Tianjin, China). 1-Ethyl-3-(3-dimethylaminopropyl)carbodiimide (EDC), and *N*-hydroxysuccinimide (NHS) were obtained from Alfa Aesar (Tianjin, China). Disodium of 9,10-anthracenediyl-bis(methylene)dimalonic acid (ABMD) was purchased from Sigma Chemical Company. 2′,7′-Dichlorofluorescein-diacetate (DCFH-DA), was purchased from Sigma-Aldrich (St. Louis, MO, USA). The mouse breast cancer cell line (4T1 cells) and the mouse normal lung epithelial cell line (TC-1 cells) were purchased from Shanghai AOLU Biological Technology Co., Ltd. Female Balb/c mice (4–6 weeks, 18–20 g) were purchased from Shandong University Laboratory Animal Center. All the chemicals were used without further purification. Ultrapure water (18.2 MΩ cm) was used throughout the experiments.

Transmission electron microscopy (TEM) was carried out on a HT7700 electron microscope (Hitachi, Japan). UV-Vis absorption spectra were measured on a pharmaspec UV1700 UV-Vis spectrophotometer (Shimadzu, Japan). Fluorescence spectra were obtained with FLS-980 Edinburgh fluorescence spectrometer with a xenon lamp. The absorbance of formazan was measured with a microplate reader (Synergy 2, Biotek, USA) in the MTT assay. Confocal fluorescence imaging studies were performed with a TCS SP5 confocal laser scanning microscopy (Leica, Germany).

### Synthesis of amino-functionalized cobalt oxyhydroxide nanoflakes (CoOOH-NH_2_)

CoOOH was synthesized following previous method. Typically, 30 mL of NaOH (0.8 M) and 30 mL of NaClO (0.1 M) were added to a 30 mL of CoCl_2_ (10 mM) solution, followed by 10 min sonication. The precipitate was then washed with water for three times and dried in oven. The as synthesized neutralized CoOOH nanosheets (10 mg) were dissolved into 5 mL anhydrous DMF, and 40 μL APTES was added to the mixture for 12 h at 80 °C under continuous mechanical stirring to activate the amino group. The product was washed twice with ethanol and water, respectively, and finally dispersed in 2 mL water to store at room temperature. The amino groups were measured by ninhydrin: 1% of ninhydrin was added to 1 mL CoOOH-NH_2_ or supernatant of CoOOH-NH_2_, and then brought to boiling for 30 min.

### Preparation of CoOOH–Ce6 nanophotosensitizer

EDC (20 μmol) and NHS (20 μmol) were mixed with Ce6 (2 μmol) in buffer in the dark for 30 min. Afterwards, 400 μL of the previously prepared CoOOH-NH_2_ solution was added dropwise to the solution, the reaction was stirred in the dark for 24 h. After washing three times with ethanol, the supernatant was collected, and the precipitate was washed with water for three times. The precipitate was finally dispersed in 2 mL of water.

### Quantification of the Ce6 on the nanophotosensitizer

Ethanol solutions of different concentrations Ce6 (50 mM, 100 mM, 150 mM, 200 mM, 250 mM, 300 mM, 350 mM, 400 mM, 450 mM, 500 mM) were prepared, and the fluorescence intensity (excitation wavelength: 405 nm, emission wavelength: 660 nm) was measured. The standard curve was made by the fluorescence data.

### Ce6 release test

Different concentration of GSH (0.5 mM, 1 mM, 2 mM, 4 mM, 6 mM, 8 mM) were added to the solution of CoOOH–Ce6 (20 μL, 1 mg mL^−1^) respectively, and then stirred for 24 h at room temperature. The fluorescence intensity was measured as the same procedure above. The CoOOH–Ce6 of supernatant was measured the fluorescence intensity.

### The ^1^O_2_ detection experiment

ABMD was selected as an indicator for the detection of singlet oxygen. ABMD reacts with singlet oxygen to decrease absorbance of ABMD. Three replicates of the same concentration of photosensitizer Ce6 were taken and ABMD was added to each of the three solutions. The 655 nm wavelength laser was irradiated for 0 min, 15 min and 30 min, and the change in ABMD absorbance was observed and determined. Then three replicates of the same concentration of nanophotosensitizer were taken. 0, 10 μL and 20 μL of different amounts of 5 mM GSH were added, respectively, and ABMD was added to the three solutions. The laser of 655 nm wavelength was irradiated for 30 min. The change in UV absorption of ABMD was measured.

### Confocal fluorescence imaging

The 4T1 cells and TC-1 cells were cultured in RPMI-1640 culture medium containing 1% of double antibodies and 10% of serum at 37 °C in 5% CO_2_ atmosphere. They were inoculated into confocal dishes after a period of incubation. The cells were incubated for 12 h until they were adherent, and then RPMI-1640 medium was discarded, and CoOOH–Ce6 (40 μL, 1 mg mL^−1^) nanophotosensitizer was added thereto. The cultivation was continued for 8 h. Then RPMI-1640 medium with the nanophotosensitizer was discarded and excess nanophotosensitizer that did not enter the cells was washed away with PBS. The cells were examined with CLSM with 405 nm excitation.

### Cell MTT assay

4T1 cells were seeded in a 96-well plate and further cultured for 24 h. After removing the culture medium, cells were incubated with different concentrations of CoOOH–Ce6 (20, 40, 80 μg mL^−1^) nanosystem at 37 °C for 6 h. Afterwards, the cells were replaced with 200 μL of fresh medium and further cultured for 48 h. The cells without any treatment as the control group were incubated for 48 h at 37 °C. In addition, 200 μL MTT solutions (0.5 mg mL^−1^ in PBS) were added to each well and incubated for 4 h. The formazan crystals formed by viable cells were solubilized in 200 μL dimethylsulfoxide and then the absorbance value was measured at 490 nm with microplate reader. To evaluate different intensity of laser for 4T1 cells, MTT assays in 4T1 cells with each sample were performed for 6 h at 37 °C, respectively. The cells were replaced under the 655 nm laser with different intensity (25, 50 mW cm^−2^). After 48 h incubation, MTT assays were carried out as the same procedure described above.

### Animal studies

All animal experiments were carried out and following the Principles of Laboratory Animal Care (People's Republic of China). Specific pathogen-free (SPF) female Balb/c mice were used in accordance with the guidelines of the principles of the Animal Investigation Committee approved by Biology Institute of Shandong Academy of Science, China. Murine tumor xenograft models were generated by the subcutaneous injection of 1 × 10^6^ 4T1 cells in PBS (150 μL) into the flank of female mice (4–6 weeks old, ∼20 g). When the tumor volume reached 30–50 mm^3^, the mice (*n* ≥ 4 per group) were injected with CoOOH–Ce6 (2 mg mL^−1^, 50 μL) (group 1), CoOOH–Ce6 (2 mg mL^−1^, 50 μL) (group 2), Ce6 (0.8 μM, 50 μL) (group 3), PBS (50 μL) (group 4), and nothing (group 5) for the first day, and the group 1, 3, 5 were irradiated with laser (655 nm, 50 mW cm^−2^, 30 min). Then, these materials were intratumorally injected into the mouse tumor. In addition, these materials were injected at day 1, 3 and 5 for three times. Tumor volumes and body weights in each group were monitored for 14 days. By recording the tumor size of the mice within 14 days, the efficacy of the treatment in the control and experimental groups were evaluated. The tumor volume (*V*) was determined by measuring length (*L*) and width (*W*), and calculated as *V* = *L* × *W*^2^/2. The relative tumor volumes were calculated for each mouse as *V*/*V*_0_ (*V*_0_ was the tumor volume when the treatment was initiated).

## Results and discussion

### Synthesis and characterization of the nanophotosensitizer

**Fig. 1:**
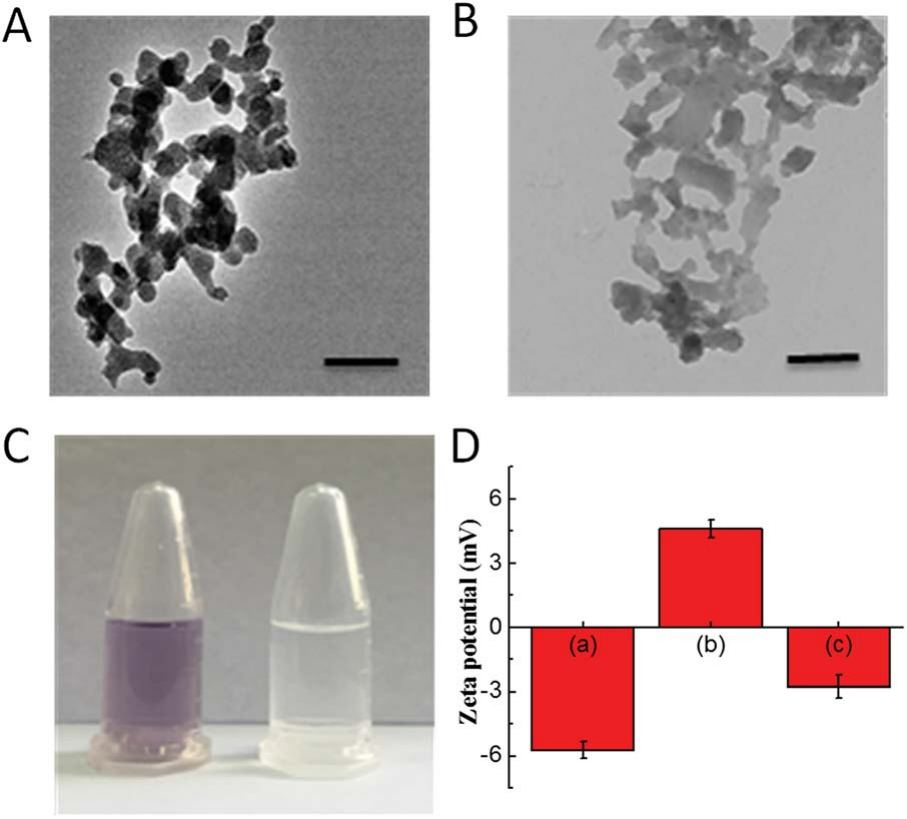
Characterization of the nanomaterials. TEM of CoOOH (A) and nanophotosensitizer (B). Scale bars are 200 nm. (C) The verification of amination of CoOOH-NH_2_ with ninhydrin experiment. On the left is CoOOH-NH_2_, and on the right is supernatant. (D) Zeta potential of each step of the modification: (a) CoOOH; (b) CoOOH-NH_2_; (c) nanophotosensitizer.

The CoOOH nanosheets were synthesized using a previously reported cobalt chloride oxidation method.^27^ APTES was employed to functionalize CoOOH nanosheets, and the final nanophotosensitizer was prepared by decorating Ce6 onto the aminated CoOOH through an amino bond. According to the transmission electron microscope (TEM) images (Fig. 1A and B), the CoOOH nanosheets displayed an average size of approximately 80 nm and the structure of CoOOH did not change obviously after the two-step modification. From the ninhydrin experiment as shown in Fig. 1C, it was clear that the precipitate turned blue-purple while no color change can be observed from the supernatant, indicating that the CoOOH has been successfully aminated and no amino group was present in the supernatant. Zeta potential of these materials was also tested (Fig. 1D). The potential of the CoOOH nanosheets was approximately −5.7 ± 0.4 mV after surface amination, and the potential became +4.6 ± 0.4 mV due to the successful modification of the positively charged group. After the photosensitizer Ce6 was attached, the potential reduced to −2.8 ± 0.5 mV, demonstrated the successful synthesis of CoOOH–Ce6 nanophotosensitizer. The amount of Ce6 molecules attached to CoOOH was quantified through a drawn standard curve. After measuring the fluorescence intensity of the supernatant, the Ce6 content of the final nanophotosensitizer was calculated to be 410 nmol mg^−1^ (Fig. S1 and S2†). To test the GSH responsive degradation of CoOOH, we performed the TEM imaging and observed the morphology change of the GSH-degraded CoOOH nanosheets. As shown in Fig. S5,† the CoOOH nanosheets were degraded after incubated in GSH solution. Moreover, color changes of the CoOOH nanosheets after incubated in GSH solution were recorded at different times. Fig. S6† indicated that the CoOOH nanosheets gradually degraded after incubation with GSH.

### Fluorescence recovery of Ce6 in the nanophotosensitizer

Because the photosensitizer Ce6 was covalently attached onto the CoOOH, the quenching effect resulted an ‘OFF’ state and the nanophotosensitizer cannot generate ^1^O_2_ under irradiation.

**Fig. 2:**
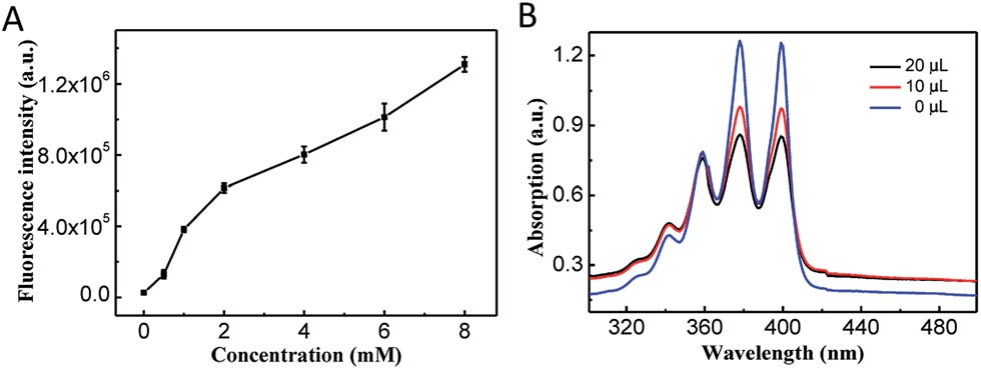
(A) Fluorescence intensity of Ce6 after CoOOH–Ce6 reacted with different concentrations of GSH. (B) The absorbance values of ABMD were measured after different amounts of GSH were added and irradiated with 655 nm laser.

After reacted with GSH, the CoOOH nanosheets can be reduced into Co^2+^, and Ce6 can thus be reactivated and used for cancer cell selective PDT. To demonstrate the reaction between GSH and CoOOH as well as the GSH triggered fluorescence recovery, the fluorescent spectra of Ce6 were recorded after CoOOH–Ce6 reacted with different amounts of GSH. As shown in Fig. 2A, the fluorescence intensity of the solution increased linearly with the increase of GSH content, which was attributed to the degradation of CoOOH. It suggested that the current nanophotosensitizer could be re-activated by GSH.

### Detection of the reactive oxygen species production

ABMD (disodium salt) was used as an indicator for detecting ^1^O_2_. Since ABMD can react with ^1^O_2_ to produce endoperoxide, which will cause the absorbance intensity decrement of ABMD.^31^ Ce6 was irradiated with laser at different times, and it was verified that the photosensitizer Ce6 can produce more ^1^O_2_ with the extension of the irradiation time (Fig. S3†). Therefore, the ^1^O_2_ produced by CoOOH–Ce6 under different GSH was investigated. As shown in the Fig. 2B, with the amount of GSH increased from 0 μL to 10 μL to 20 μL, the UV-Vis absorption of ABMD obviously decreased under laser irradiation, indicating that GSH degraded CoOOH nanosheets, and reactivation of Ce6 effectively produced ^1^O_2_. Intracellular ROS production was detected with DCFH-DA^32^ to demonstrate the nanophotosensitizer can be used for cancer cell selective PDT. As shown in Fig. S7,† the strong green fluorescence indicated that ROS can be produced when irradiated with NIR laser. These results demonstrated that the nanophotosensitizer could be used for GSH responsive PDT.

### Therapeutic effect of the nanophotosensitizer in living cells

**Fig. 3:**
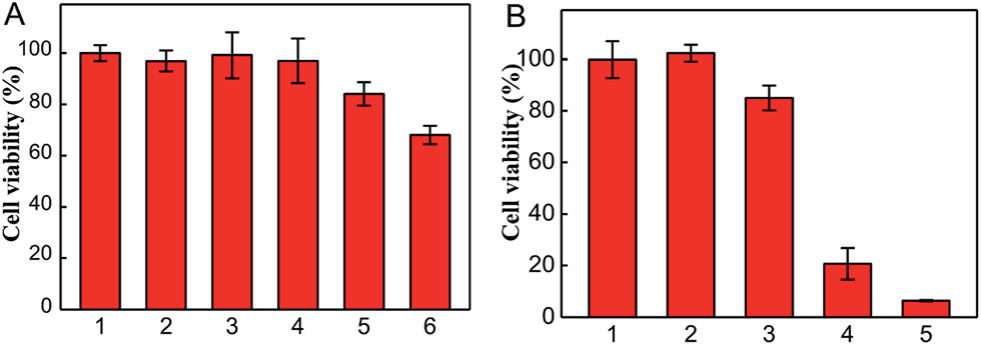
(A) The cell viabilities of 4T1 cells after different treatments: without any treatment (1), irradiated with 655 nm laser at the intensity of 25 (2) and 50 (3) mW cm^−2^ for 30 min, respectively; incubated with the CoOOH–Ce6 (20 μg mL^−1^ (4); 40 μg mL^−1^ (5); 80 μg mL^−1^ (6)) without laser irradiation. (B) Cell viabilities of 4T1 cells after different treatments: blank control (1), Ce6 (38 μg mL^−1^) only (2), CoOOH–Ce6 (40 μg mL^−1^) only (3), Ce6 + laser (4), CoOOH–Ce6 + laser (5), the intensity was 50 mW cm^−2^.

For confirming the optimum laser parameters and further investigating the PDT effect of the nanophotosensitizer in living cells, MTT experiments were carried out.^33^ 4T1 cells were treated with different intensities (25, 50 mW cm^−2^) of laser irradiation alone and different amounts of CoOOH–Ce6 (20, 40, 80 μg mL^−1^). As shown in Fig. 3A, cells showed negligible cell viability decrement under laser irradiated alone, which means 655 nm laser irradiation cause little harm to cells even after 30 min. With the increase of the nanophotosensitizer concentration from 20, 40, to 80 μg mL^−1^, the cell viability decreased from 96% to 83% and 68%, respectively. Therefore, 50 mW cm^−2^ laser intensity and 40 μg mL^−1^ CoOOH–Ce6 were chosen for the following experiments. To demonstrate the effect on the survival rate of cancer cells for the NIR irradiated CoOOH, different concentrations of CoOOH nanosheets were added to 4T1 cells and incubated for 6 h. After illuminated with a 655 nm laser for 30 minutes, the cells were further incubated for another 48 h and the survival rate of the treated cells was determined. Fig. S4† showed that no obvious suppressing effect was observed for the selected concentration (40 μg mL^−1^). Next, MTT assay was further carried out to test the therapeutic effect of the nanophotosensitizer. Fig. 3B showed that the cell survival rate was high, when incubated with Ce6, CoOOH–Ce6 or laser irradiation alone. While the cell viability of the NIR irradiated CoOOH–Ce6 group decreased sharply to 8.3%, indicating that the nanophotosensitizer can significantly enhance the therapeutic effect of cancer PDT owing to the GSH clearance capability and ^1^O_2_ generation. These results confirmed that the nanophotosensitizer was a promising platform for highly efficient PDT.

**Fig. 4:**
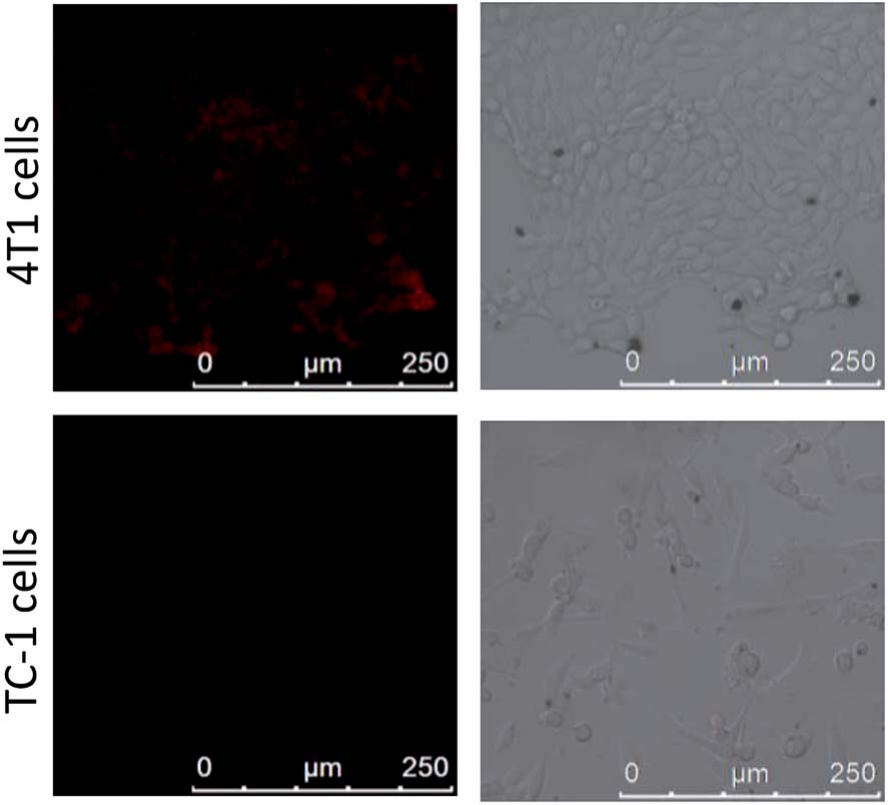
Confocal imaging of 4T1 cells and TC-1 cells after incubated with CoOOH–Ce6 nanophotosensitizer (40 μg mL^−1^) for 8 h.

To determine the selectivity of the nanophotosensitizer for cancer cells, nanophotosensitizer (40 μg mL^−1^) was employed to incubate with 4T1 cells and TC-1 cells. The fluorescence intensity of Ce6 was evaluated by CLSM after 8 h incubation. As shown in Fig. 4, the fluorescence intensity of Ce6 was brighter in cancer cells than in normal cells. This is because the GSH levels in tumor cells are overexpressed, thus more CoOOH can be degraded, resulted in higher fluorescence recover rate and stronger fluorescence intensity, which is critical for cancer PDT with high selectivity.

### Evaluation of the nanophotosensitizer for PDT *in vivo*

**Fig. 5:**
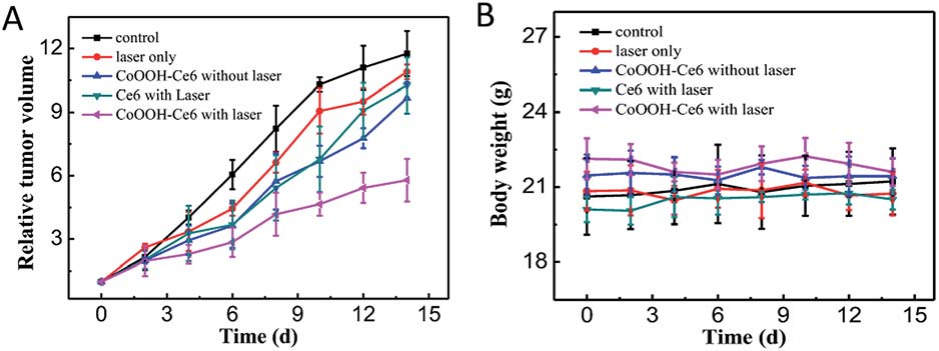
Tumor growth curves (A) and mouse body weight curves (B) of different treatments of tumor-bearing mice: PBS only, laser only, CoOOH–Ce6 without laser, Ce6 with laser, CoOOH–Ce6 with laser.

Before evaluating the PDT effect *in vivo*, the haemolytic effect was determined (Fig. S8†). The results suggested that the nanophotosensitizer had non-hemolytic effect toward the red blood cells of mice, indicating that CoOOH–Ce6 can be used for *in vivo* PDT. 4T1 tumor-bearing mouse models were used to demonstrate that the synthetic nanophotosensitizer have the capacity for treating tumors *in vivo*. The 4T1 cells were first injected into the armpit of the mice. The tumor-bearing mice were divided into five groups for different treatments: control (PBS only), laser irradiation only, Ce6 with laser irradiation, CoOOH–Ce6 only and CoOOH–Ce6 with laser irradiation. After 5 days, the nanophotosensitizer CoOOH–Ce6 were dispersed into the PBS buffer solution (2 mg mL^−1^, 50 μL) and intratumorally injected into the mouse tumor at day 1, 3 and 5 for three times. The 655 nm laser with a power density of 50 mW cm^−2^ was used to irradiate the tumor area for 30 min each time. By recording tumor volumes and body weights of the mice within 14 days, the efficacy of the treatment in the control and experimental groups were evaluated. The experimental results from Fig. 5A indicated that the treatments with only laser irradiation and with the nanophotosensitizer alone had no tumor inhibition capability. Treatment with Ce6 alone could inhibit tumor growth under laser irradiation, which showed that the photosensitizer Ce6 can generate ^1^O_2_ to kill tumor cells under illumination. However the efficiency remains poor, and the resistance was mostly date from the over-expressed GSH. Compared with the control group, the nanophotosensitizer we designed exhibited more pronounced inhibitory effects on tumors under light irradiation. These results suggested that the nanophotosensitizer had better tumor therapeutic effect, demonstrating that CoOOH is a promising platform to enhance the PDT efficiency due to the GSH elimination. As shown in Fig. 5B, there was no significant change in the body weight of the mice during treatment, further indicating that the nanophotosensitizer was non-toxic. Afterwards, H&E staining assays on each group of mice. No obvious lesions can be observed in all tissues and organs (heart, liver, spleen, lung and kidney) (Fig. S9†). This further demonstrated that CoOOH–Ce6 nanophotosensitizer had good biocompatibility and was promising for cancer PDT.

## Conclusions

In summary, we designed and synthesized a CoOOH-based smart nanophotosensitizer for PDT of cancer. The nanophotosensitizer was synthesized by directly decorating photosensitizer Ce6 onto aminated CoOOH nanosheets *via* amide bonds. This nanophotosensitizer was quenched in normal cells and it showed little damage to normal cells. When entering cancer cells, CoOOH nanosheets can be degraded by the overexpressed GSH in cancer cells, resulting in the release of Ce6 molecules and the effectively production of ^1^O_2_ under laser excitation. At the same time, the consumption of GSH reduced the tolerance of cancer cells to ^1^O_2_. Therefore, this nanophotosensitizer exhibited good selectivity and enhanced cancer treatment effects, which were confirmed both *in vitro* and *in vivo*. We anticipate that the nanophotosensitizer can provide new insights for cancer PDT.

## Conflicts of interest

There are no conflicts or competing financial interest.

## Supplementary Material

RA-008-C8RA08549H-s001
