# Supplementary material for: A GSH-responsive nanophotosensitizer for efficient photodynamic therapy
Source: RSC Adv. 2018 Dec 19;8(74):42374–9. doi: 10.1039/c8ra08549h (PMC9092154; doi:10.1039/c8ra08549h)
Supplement: RA-008-C8RA08549H-s001 [file RA-008-C8RA08549H-s001.pdf]

Electronic Supplementary Material (ESI) for RSC Advances.
This journal is © The Royal Society of Chemistry 2018

Supplementary Information

A GSH-responsive nanophotosensitizer for efficient photodynamic therapy

Wei Pan,‡ Mingwan Shi,‡ Yanhua Li, Yuanyuan Chen, Na Li* and Bo Tang

College of Chemistry, Chemical Engineering and Materials Science, Key Laboratory of Molecular and Nano Probes, Ministry of Education, Collaborative Innovation Center of Functionalized Probes for Chemical Imaging in Universities of Shandong, Institute of Molecular and Nano Science, Shandong Normal University, Jinan 250014, P. R. China.

E-mail: lina@sdnu.edu.cn

‡These authors contributed equally to this paper.

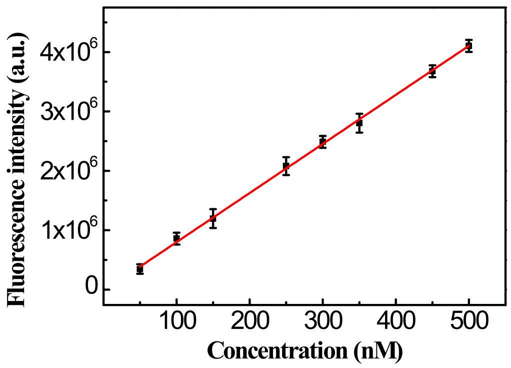

Fig. S1 Fluorescence intensity standard linear curve of Ce6.

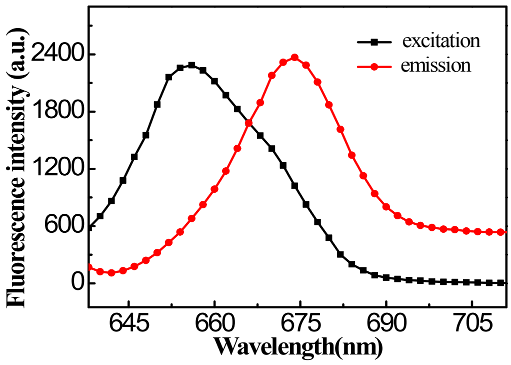

Fig. S2 Excitation and emission wavelengths of Ce6.

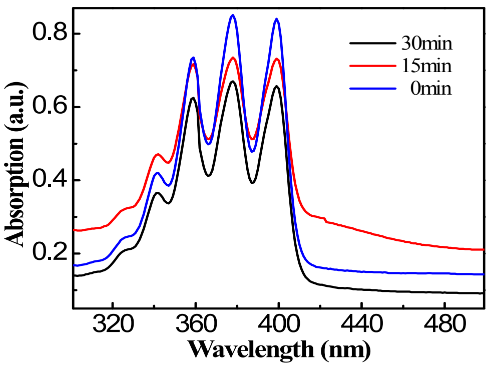

Fig. S3 The absorbance values of ABMD were measured by irradiating the photosensitizer Ce6 with 655 nm laser irradiation for 0 min, 15 min, and 30 min, respectively.

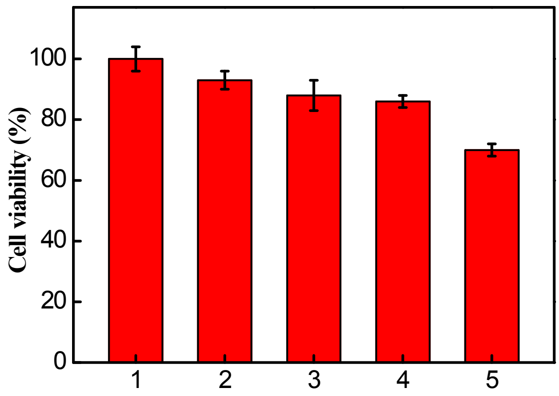

Fig. S4 The cell viability of 4T1 cell after different treatments: PBS as control (1), incubated with the CoOOH (20 μg•mL^{-1} (2); 40 μg•mL^{-1} (3); 60 μg•mL^{-1} (4) 80 μg•mL^{-1} (5)) with 655 nm laser at the intensity of irradiation 50 mW•cm^{-2} for 30 min.

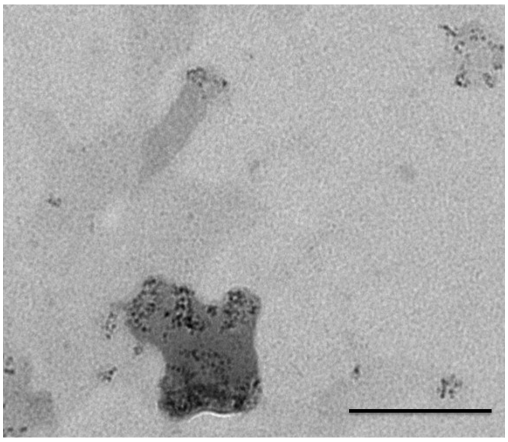

Fig. S5 TEM image of the CoOOH nanosheets after incubated in GSH solution (scale bar : 100 nm)

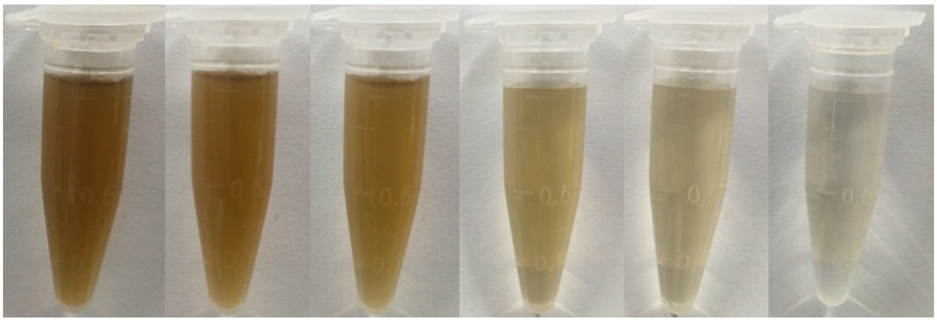

Fig. S6 Color changes of the CoOOH nanosheets after incubated in GSH solution at different time points.

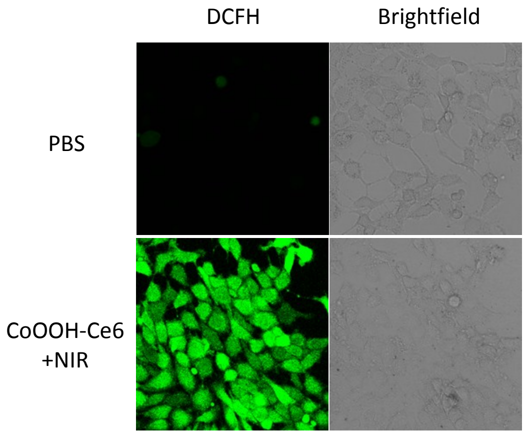

Fig. S7 Confocal imaging of 4T1 cells after incubated with different treatments.

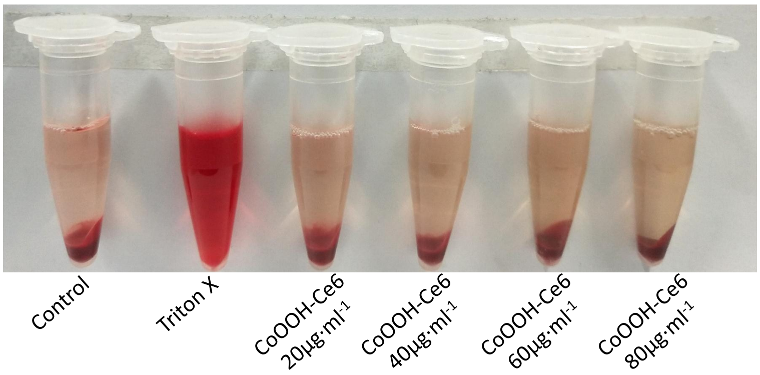

Fig. S8 Hemolytic assay of the CoOOH-Ce6.

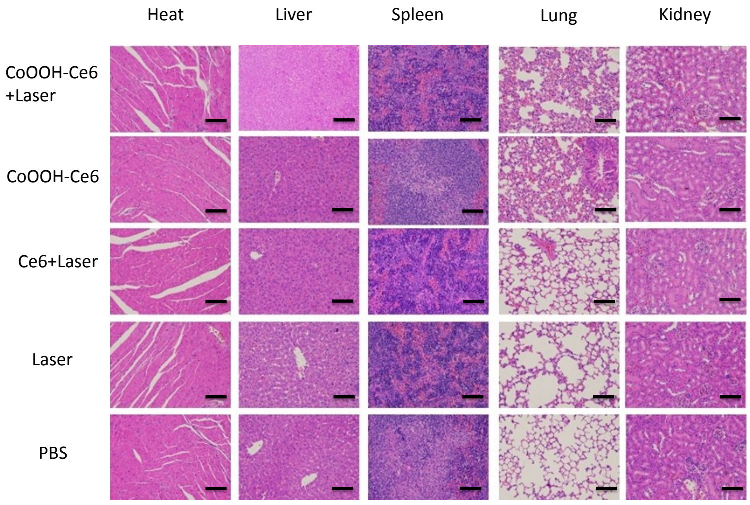

Fig. S9 Biocompatibility of the nanoparticles. H&E staining images (200×, scale bars are 100 μm.) of five major organs (heart, liver, spleen, lung, and kidney) at 7 day after different treatment groups: PBS only; laser only; Ce6 with laser; CoOOH without laser; CoOOH-Ce6 with laser.
